# The Physicochemical Properties and Melting Behavior of Ice Cream Fortified with Multimineral Preparation from Red Algae

**DOI:** 10.3390/foods12244481

**Published:** 2023-12-14

**Authors:** Joanna Markowska, Agnieszka Tyfa, Anna Drabent, Artur Stępniak

**Affiliations:** 1 Prof. Waclaw Dabrowski Institute of Agricultural and Food Biotechnology—State Research Institute (IBPRS-PIB), Department of Refrigeration Technology and Technique, Marszalka J. Pilsudzkiego 84 Avenue, 92-202 Lodz, Poland; agnieszka.tyfa@ibprs.pl (A.T.); anna.drabent@ibprs.pl (A.D.); 2 Unit of Biophysical Chemistry, Department of Physical Chemistry, Faculty of Chemistry, University of Lodz, Pomorska 163/165 Street, 90-236 Lodz, Poland; artur.stepniak@chemia.uni.lodz.pl

**Keywords:** ice cream, meltdown, thermal fluctuations, multimineral preparation, sensory analysis, nutrient density

## Abstract

Ice cream is a popular frozen food consumed worldwide throughout the year. However, as a thermally unstable product, it requires proper cold chain management. Thermal fluctuations alter the physicochemical properties of ice cream and reduce its quality. This study was conducted to evaluate the physicochemical and sensory properties of ice cream containing different amounts (0.5; 0.8; 1.0%) of a multimineral preparation from Atlantic red algae. The effect of thermal shock on the quality of ice cream after preparation and 90 days of frozen storage was studied. The addition of a multimineral component slightly increased the freezing and glass transition temperatures of the ice cream. The overrun of the ice cream ranged from 48.55 to 52.78% and decreased with the frozen storage time, but the samples with 0.8 and 1.0% mineral content had the most stable overrun in terms of storage time and thermal fluctuations. Ice cream stored for both 7 and 90 days showed a similar melting behavior, although a shift in the melting curves was observed after long frozen storage. The samples exposed to the thermal treatment had lower melting rates by 39.2–59.9% and 55.2–65.4% for 7-day and 90-day stored ice cream, respectively. The hardness parameters of the ice cream did not change significantly under the conditions applied, so the fragility of the ice cream and its fluffiness did not seem to be affected. The organoleptic evaluation showed that ice cream with a mineral content of 0.8% was the most acceptable in terms of taste, texture, and overall acceptability. The applied mineral and sucrose content ratios did not alter the main physicochemical and organoleptic parameters, but significantly affected the nutrient density of the ice cream.

## 1. Introduction

Ice cream is considered to be a specific food product that requires the maintenance of low temperatures throughout the production chain, i.e., freezing, frozen storage, and consumption in the frozen state. The typical ice cream formulation consists of several ingredients: lipids, proteins, hydrocolloids, carbohydrates, stabilizers, and emulsifiers dispersed in the formulation matrix. Further processing involves freezing the formulations with simultaneous mixing and aeration, which is responsible for the development of the ice cream structure. This aerated structure is stabilized by matrix viscosity, the presence of ice crystals and air cells, partially coalesced fat globules, and stabilizing emulsifiers.

The sensory quality of ice cream is related to its physicochemical properties, including overrun, air bubble size, and ice crystal morphology, which are crucial for the development of ice cream texture [1]. The nature and proportions of the components selected for ice cream formulation preparation are also important as they modify various ice cream parameters. The most cited components are dairy ingredients and their substitutes for vegan ice cream production (e.g., inulin), milk and non-milk fat content, presence of vegetable oils, flavorings, and stabilizers, which have been observed to influence the textural and sensory attributes of ice cream [2,3,4]. In general, the textural quality perceived by the consumers is better in ice cream containing a significant amount of ice crystals with an average size between 20 and 75 µm, preferably below 55 µm [5]. Larger ice crystals increase the sensation of iciness and sandy texture [6]. Similarly, the lightness of ice cream depends on the air cell size, which varies between 30 and 150 µm in good quality products [7]. Due to its specific characteristics, ice cream is thermodynamically unstable and sensitive to temperature changes, especially during transport and storage, so any change in external conditions will affect the final quality of the ice cream. Unstable conditions favor the melting of ice and its subsequent recrystallization, often forming ice crystals of larger average size [8]. To prevent this phenomenon, ice cream formulations are supplemented with functional substances characterized by a cryoprotective effect—hydrocolloids (polysaccharides, proteins) [9]. Water-binding ingredients increase the viscosity of ice cream mixes and limit ice recrystallization. They also improve the ice cream’s resistance to thermal stress and help maintain the ice cream’s shape during melting and shrinking during storage [10]. Typical hydrocolloids used in ice cream production have different modes of action and, therefore, different cryoprotective effects. Agar, κ-carrageenan, and locust bean gum reduce the degree of ice recrystallization and improve the internal microstructure of ice cream [11]. These are often used as stabilizers. The addition of alginate, gelatin, and guar gum aims to increase the viscosity of the ice cream mix, prevent syneresis, control melting, and limit product dehydration and, therefore, ice cream shrinkage.

Microalgae preparations are used globally as an additive in ice cream production to improve the physicochemical properties and to modify the sensory characteristics and appearance of the ice cream [12,13]. The usage of multimineral preparation derived from Atlantic red algae of the Corallinacea family (calcareous marine algae *Lithothamnion* sp.) as an ingredient in dairy ice cream production seems promising. The health-promoting features and technological functionality of a chosen multimineral preparation have been proven in bread, pastries, yogurt, and chocolate production [14,15,16,17]. Therefore, we have considered the preparation of a mineral-fortified ice cream with properties similar to those of standard dairy ice cream and with a potentially beneficial effect on the human diet in terms of nutrient uptake. The objective of our research was to prepare mineral-fortified dairy ice cream and to evaluate its stability in terms of structural, physicochemical, and organoleptic changes after exposure to thermal fluctuations and frozen storage.

## 2. Materials and Methods

### 2.1. Ice Cream Mix Preparation

Ice cream mixes were formulated to contain different amounts of the ingredients listed in Table 1. Skim milk powder (Mlekovita, Wysokie Mazowieckie, Poland), sucrose (Diamant, Miejska Górka, Poland), and milk fat (SM Mlekpol, Grajewo, Poland) were purchased from local wholesalers. The integrated blend of emulsifier and stabilizer was purchased from Palsgaard A/S (Juelsminde, Denmark). The marine algae multi-mineral source was purchased from Marigot Group Cork (Cork, Ireland) and its mineral composition is given in Table S1 (see Supplementary Materials).

Skim milk powder, milk fat, sucrose, emulsifier–stabilizer, and multi-mineral powder preparation were dissolved in tap water and pasteurized at 80.0 ± 1.0 °C for 15 min under continuous stirring (80 rpm) using Achiever 5000 Stirrer (OHAUS Europe GmbH, Nänikon, Switzerland). The pasteurized formulations were cooled and homogenized (Homogenizer CAT, Model Unidrive X 1000 MPW-120, Staufen, Germany) at 14,000 rpm for 15 min. Homogenized ice cream mixes were aged at 4.0 ± 1.0 °C for 20 h before further processing. 

### 2.2. Ice Cream Preparation (Freezing Conditions and Storage)

The aged ice cream mixes were frozen in a Please change the (model RQ18 T, Guangzhou Guangshen Electric Produce Co., Ltd., Guangzhou, China) in the automatic model so that the temperature of the ice cream mass after the extrusion was around −5.0 ± 0.5 °C. The ice creams were collected in 250 mL plastic containers, then subjected to shock freezing (Arktic, Hendi BV, De Klomp, The Netherlands) and stored at −35.0 °C until further analysis. Ice cream samples were prepared in triplicate.

### 2.3. Analyses

#### 2.3.1. Physicochemical Analysis

Selected physicochemical parameters of aged ice cream mixes were studied. The dry matter content of the ice cream mixes was measured using the MA40 Moisture Analyzer (Sartorius, Göttingen, Germany). The water activity (a_w_) of the ice cream mixes was measured using the water activity meter AquaLab4 (Decagon Devices Inc., Pullman, WA, USA). The pH of the ice cream mixes was measured using the pH-meter CP-505 (Elmetron, Zabrze, Poland). Protein content was determined by the Kjeldahl method [18]. 

#### 2.3.2. Mix Viscosity

The rheological properties of the aged ice cream mixes were determined using an RST-CC Rheometer (AMETEK Brookfield, Middleborough, MA, USA) equipped with Rheo3000 software. All the analyses were carried out using a controlled shear rate (CSR) profile via a cylindrical measuring system. For the experiment, a rotational ramp measurement block was selected with the following parameters: Spindle shear rate (*v*_r_): 150–600 s^−1^; time: 300 s; measuring points: 60; temperature: 5.0 °C. The obtained data were fit using Ostwald de Waele and Herschel–Bulkley models:

σ = k × γ^n^ (Ostwald de Waele)

σ = σ_0_ + k × γ^n^        (Herschel-Bulkey)

where *σ* is the shear stress [Pa], *σ*_0_ is the yield stress [Pa], γ is the shear rate [s^−1^], k is the consistency coefficient [Pa *×* s^n^], and n is the flow behavior index. In addition, the viscosity of the ice cream mixes was determined at a shear rate of 350 s^−1^. The analyses were performed in triplicate.

#### 2.3.3. Ice Cream Mix Freezing

The dynamics of static freezing of ice cream mix were studied. In total, 100 mL of ice cream mix was placed in a cylindrical aluminum container (height 50 mm, diameter 51 mm) with a lid with an equipped thermocouple probe and stored in a freezer at −30.0 °C. The freezing process was monitored by temperature changes recorded by a single-channel HOBO UX100-014 M thermocouple data logger with a K-type thermocouple probe (Onset Computer Corp, Bourne, MA, USA). The data obtained were analyzed using HOBOware software Version 3.7.23 (Onset Computer Corp, Bourne, MA, USA) and then presented as a function of time and considered as freezing curves. Afterwards, the freezing temperatures (*Tf*) of the samples were determined from the curves, as described by Rahman et al. (2002) and Bainy et al. (2015) [19,20]. The highest temperature of the flat part of the curve was regarded as the freezing temperature. The measurements were performed in triplicate.

#### 2.3.4. Glass Transition Measurement

The ice cream glass transition temperature (*Tg*) was determined by differential scanning calorimetry (DSC) using a Linseis Chip-DSC 100 differential scanning calorimeter (Linseis Inc., Selb, Germany). Samples weighing 16 mg were placed in the 20 µL aluminum crucibles and stored at −35 °C. Then, the samples were placed in a liquid nitrogen cooled calorimeter and kept until the temperature of the samples reached >−70 °C. The measurement was started by recording the thermogram in the range of −70 °C to 15 °C so that the glass transition and melting process could be observed. The heating rate was 10 degrees/min. The glass transition was determined using the half-height method. The midpoint was regarded as the value on the *y*-axis (heat flow) halfway between the calculated start and end of the glass transition area. The temperature on the *x*-axis corresponding to the heat flow value on the y-axis was given as the glass transition (*Tg*). The analysis was based on the points of intersection of the tangents where slight variations in the angles of the tangents to the thermogram can cause changes in the values obtained. All the experiments were performed in triplicate.

#### 2.3.5. Texture

The texture analyzer CT3 TA (AMETEK Brookfield, Middleborough, MA, USA) was used to determine the hardness [N] and adhesiveness [mJ] of ice cream according to the procedure described by Tiwari et al. (2015) with modifications [21]. Ice cream samples (250 mL) stored for 7 days at −25.0 °C were transferred to the freezer at a temperature of −10.0 °C and stored overnight. Afterward, the Texture Profile Analysis (TPA) was conducted using a 4 mm stainless steel cylinder probe TA44. For the TPA test, the following conditions were applied: penetration distance 15 mm, force 0.5 N, probe speed during penetration 3.3 mm × s^−1^, probe speed post-penetration 3.3 mm × s^−1^. Obtained data were analyzed using TexturePro CT V 1.2 Build 9 software. All experiments were carried out in triplicate.

#### 2.3.6. Overrun

The overrun (OR) of ice cream was calculated based on the weight difference between fixed volumes of ice cream mix and frozen ice cream. Overrun measurements were performed in triplicate. The calculations were made based on the following equation:

$$\mathrm{OR}\;\left(\%\right)=\frac{\mathrm{mix}\;\left(g\right)-\mathrm{ice}\;\mathrm{cream}\;\left(g\right)}{\mathrm{ice}\;\mathrm{cream}\;\left(g\right)}\times 100\%$$

#### 2.3.7. Meltdown Test

The meltdown test was proceeded according to the methods described in the literature with modifications [22,23,24]. Ice cream stored for 7 days at −25.0 °C was taken for the experiment. A cylindrical ice cream sample of around 20 g was cut and placed on a metal wire mesh suspended over a glass beaker on a scale to monitor the melting behavior at ambient temperature (22.0 ± 1 °C). The weight of the molten ice cream was collected in the beaker and measured every 10 min for 90 min. The time of the first droplet dripped was considered as an induction time. Obtained results were presented as the percentage of ice cream drip-off using the following equation:

drip-off (%) = (m_d_/m_s_) × 100%,

where m_d_ is the weight of the ice cream drip-off (g), and m_s_ is the weight of the ice cream sample (g). Afterward, the drip-off weight was plotted against the experiment time (minutes) and the range of fast melting phase range was used for the linear fitting of the slope to determine the melting rate of the ice cream. The measurements were performed in triplicate.

#### 2.3.8. Color Evaluation

Ice cream color was determined using a spectrophotometer CM-5 Konica Minolta (Konica Minolta Inc., Tokyo, Japan) according to the methodology given by the producer. The following parameters were recorded in the CIE L*a*b* space: brightness L* (range 0 to 100), redness a*, yellowness b*, degree of color saturation C* (Chroma), and color tone h (hue). The total color difference (ΔE) was calculated using the following equation:

$$\Delta E=\sqrt{\Delta L^{*}{}^{2}+\Delta a^{*}{}^{2}+\Delta b^{{*}^{2}}}$$

The base ice cream model (S0) was considered as the reference sample. Ice cream color was evaluated 7 days after freezing as well as after 90 days frozen storage.

#### 2.3.9. Temperature Fluctuations

The impact of frozen storage temperature fluctuations was studied by subjecting ice cream to a temporary heat shock procedure. The ice cream stored at −25.0 °C for 7 days was allowed to stand at ambient temperature (22.0 ± 1.0 °C) for 60 min. Then, the samples were placed back in the freezer (−25.0 °C) and stored overnight. The procedure was repeated twice. After that, ice cream samples were taken for further analysis (color, overrun, meltdown, texture). All the measurements were performed in triplicate. The experiment was proceeded both for aged ice cream and ice cream stored for 90 days.

#### 2.3.10. Ice Cream Stability after Storage

Produced ice cream was stored in the freezer at −35.0 °C for 90 days. After that time, ice cream was analyzed to verify whether any changes in color, overrun, meltdown behavior, and texture occurred. The experiment was conducted for untreated ice cream and those subjected to temperature fluctuations. 

#### 2.3.11. Sensory Analysis

Sensory evaluation of ice cream models was performed by six trained panelists according to consumer preference analysis and quantitative description analysis QDA (sense profiling). In the QDA test, the assessors evaluated several ice cream characteristics using a 9-point scale system where the attributes ranged from non-detectable (1) to very intensive (9). Three descriptors were evaluated according to individual ranges; orodispersible structure from ‘fast’ to ‘slow’, oral hardness from ‘plastic’ to ‘fragile’, and the color from ‘light’ to ‘yellow’. The ice cream was kept at −12.0 °C for 24 h before the sensory assessment. The samples of approximately 15 g were served to panelists in plastic cups. Ice cream appearance, texture, and flavor were evaluated using the following attributes: (a) appearance—color, glossy, matte, moire, granular; (b) texture—gumminess, stickiness, creaminess, fluffiness, mouthcoating, coarseness, sandiness, ice crystals, oral hardness, orodisersibility; (c) odor and flavor—smell (typical for milk), sweet, acid, milk taste, buttery taste, insipid, metallic, off-flavor. The responses were analyzed using principal component analysis (PCA) and presented graphically as the assessors’ mean.

#### 2.3.12. Nutrient Density Evaluation

Nutrient profiling was determined using several nutrient density models which consider various algorithms and nutrient sets. The chosen models included naturally nutrient-rich score (NNR), nutrient adequacy score (NAS), nutrient density score (NDS), and nutrient-rich food index (NRF) for which the calculation is widely explained in the literature [25,26,27,28,29,30,31]. In addition, the energy value of each ice cream model was calculated.

#### 2.3.13. Statistical Analysis

The data were analyzed by Statistica^®^ 10.0 PL software (StatSoft Poland Sp. z o.o., Kraków, Poland). The results from the experiments were analyzed using one-way ANOVA and Tukey’s HSD tests (α < 0.05). All the measurements were performed in triplicate.

## 3. Results and Discussion

The effect of the addition of a multi-mineral preparation to ice cream mixes on the freezing process, ice cream overrun, melting behavior, texture, and color was investigated. The selected parameters were studied after the temperature fluctuation simulation and frozen storage time (about 90 days).

### 3.1. Ice Cream Mix Characteristics

#### 3.1.1. Ice Cream Mixes Parameters

Prepared ice cream mix models (S0, S0.5, S0.8, S1.0) were characterized by a comparable dry mass content, protein content, and water activity (a_w_). The pH of the reference ice cream mix (model S0) was equal to 6.58 ± 0.04 and appeared significantly lower than for the rest of the formulations (Table 2). This was not surprising since, as stated by the manufacturer, the mineral blend in the aqueous solution has an alkaline pH. In ice cream formulations with added multi-mineral preparation, a positive correlation between its concentration and the pH was observed. Many other studies have shown that both the addition of chemical compounds and their concentration have an effect on the acidity of ice cream mixes [32,33,34].

In addition, for each of the ice cream formulations, the energy density was calculated, which was considered as the amount of the energy available from 100 g of the mix/ice cream (kcal/100 g; kJ/100 g). A gradual decrease in energy density was observed with the increasing multimineral preparation. This was explained by the decrease in the amount of sucrose, which has the greatest effect on the calculations required to determine the energy density. Although these differences were negligible when comparing the reference model and S0.5 samples, the drop in energy density in mixes with 0.8 and 1.0% of multimineral preparation seemed small but significant (Table 2).

DSC analysis of the modified ice masses showed that an increase in the percentage of mineral preparation relative to the control sample results in an increase in the melting temperature and a slight shift in the glass transition point. In all thermograms of the analyzed samples, the occurrence of endothermic peaks characterizing the melting process was noted. The melting temperature ranged from −8.1 °C (model S0) to −5.3 °C (model with 1.0% mineral preparation) (Table 3). The ∆T values were 14.1, 14.4, and 14.6 for the models S0.5, S0.8, and S1.0, respectively, which was higher than for the control sample (∆T_S0_ = 11.5). According to Alvarez et al. (2005), the temperature range can indicate the uniformity of the size distribution of the ice crystals, with a narrower range suggesting a more homogenous distribution that will melt over a smaller range of temperatures [35]. In our study, the increasing minerals concentration with simultaneous decreasing sucrose content resulted in ∆T value increases, which might indicate enhanced heterogenous ice crystals size distribution. Compared to the reference sample, a slight reduction in the enthalpy values of the melting process in the samples with minerals added was noted. This is probably related to the change in the water content that was present in the sample analyzed and subjected to the freezing process [36,37]. A similar suggestion was made in another study, where the authors associated the drop in enthalpy values with changes in freezable water content rather than the moisture content of the samples [32]. In addition, the change in the measured enthalpy of melting can be affected by the addition of minerals and, consequently, results in a change in the heat capacity of the tested samples [38,39].

In the course of the study, glass transition measurements were also carried out for the reference sample and ice masses containing the mineral preparation added at different percentage contents. The glass transition for the reference sample was observed at −47.28 ± 0.1 °C, while for the samples containing the mineral additive it ranged from −46.26 ± 0.3 °C to −46.54 ± 0.2 °C. The addition of the mineral preparation slightly increased the glass transition temperature (Tg), however, no significant differences were found between the samples tested. Despite that, a moderately positive correlation between Tg and mineral component concentration was noted. The changes in glass transition temperatures were reported in several studies, however, originating from different mechanisms of action [40,41]. This is of particular interest in terms of ice cream quality during long-term frozen storage.

#### 3.1.2. Ice Cream Mixes Rheological Behavior

**Figure 1:**
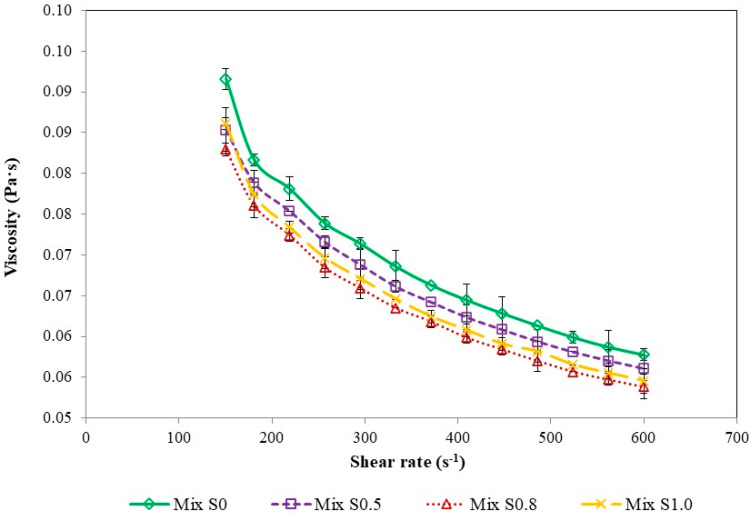
Flow behavior of ice cream mixes supplemented with multimineral preparation: 0% (mix S0), 0.5% (mix S0.5), 0.8% (mix S0.8), 1.0% (mix S1.0).

The composition and components proportions of the ice cream formulations, which are regarded as complex mixtures of water, fat, proteins, polysaccharides, stabilizers, emulsifiers, and additives, influence the rheological properties. The results obtained for ice cream mixes flow behavior are presented in Figure 1.

In general, the mixes’ flow curves showed shear thinning behavior (Figure 1) and, therefore, formulations could be classified as pseudoplastic fluids. In addition, the negative correlation between apparent viscosity and shear rate proved the typical behavior of non-Newtonian fluids. This is in agreement with the literature data on ice cream mixes rheological studies [42,43,44,45]. Ice cream with multimineral preparation addition showed a gradual viscosity decrease which differed significantly among the tested samples. The most probable reason of this phenomenon appears to be the decreasing concentration of sucrose rather than the increase of microelements content as the addition of sugar is associated with higher viscosity [46]. This has been observed in studies considering sucrose replacement with sweeteners such as stevia or dates [47,48]. The viscosity drop was also reported by Abd El-Khair et al. (2020) in ice cream mixes in which a particular content of butter oil was replaced by inulin [49]. Surprisingly, the viscosity increase was reported in ice cream supplemented with dietary fibers, macromolecular sweeteners (cornstarch hydrolyzates and oligosaccharides), proteins, protein-based fat replacers, stabilizers, hydrocolloids, and also inulin [40,50,51,52,53,54,55]. The duration of ice cream formulation aging is reported to affect the mix viscosity as well, but not always necessarily [56,57]. In this study, the values of apparent viscosity at a chosen shear rate (350.00 s^−1^) were found to be significantly lower than for the reference sample (Table 4).

The collected data show that the Herschel–Bulkley model fits better to ice cream flow behavior than the Ostwald de Waele model as the stability indices (R^2^) values were higher (Table 4). The ice cream mixes showed a non-Newtonian characteristic, exhibiting yield stress and shear thinning, i.e., during shear rate increase the decrease in apparent viscosity was noted. According to several studies, the pseudoplastic behavior of ice cream mixes have a flow index (n) below unity. The flow indices values both in Ostwald de Waele and Herschel–Bulkley models were 0.5286–0.5414 and 0.7459–0.7667, respectively, and remained in agreement with the literature data concerning various additives [58,59,60]. Surprisingly, in the samples with the addition of minerals at concentrations of 0.5% and 0.8%, a sudden and significant decrease in yield stress (*σ_0_*) was observed (Table 4). However, this effect could be within the variability of the measurements, especially since the difference between the yield stress values of the reference model and the S1.0 model was not significant. This suggests that the selected mineral concentration, together with a slightly lower sucrose concentration in the samples studied, has a negligible effect on the rheological parameters of the ice cream mixes.

### 3.2. Ice Cream Characteristics

#### 3.2.1. Ice Cream Color Profile

The color parameters of the ice cream samples are presented in Table 5. Reference ice cream (model S0) appeared to be characterized by the highest L* value and thus all samples with mineral complex addition were categorized as significantly darker. The ice cream containing 1.0% multi-mineral preparation had the lowest L* parameter, as expected. The changes in a* and b* color parameters indicate that all tested ice cream models exhibited slightly more green and more yellow color than ice cream without mineral complex. 

After 90 days frozen storage, a moderate change in ice cream color parameters was observed (Table 5). The L* parameter values of reference ice cream and ice cream with 0.5% multimineral preparation addition were lower, which indicates sample darkening. On the contrary, a higher concentration of minerals resulted in lighter ice cream color. In all ice cream samples, the decrease of a* value was noted, which suggests an intensification of green color. Ice cream storage caused redness and color saturation increase, although the changes appeared insignificant. Surprisingly, in the reference sample and ice cream S1.0 model, an opposite trend was observed.

The total color difference (ΔE) showed a significant impact of multi-mineral additive on the overall ice cream color. According to the literature data, the ΔE values can be classified into five different groups: (a) 0 < ΔE < 1—invisible color change; (b) 1 < ΔE < 2—color difference recognizable by a trained panelist only; (c) 2 < ΔE < 3.5—color difference recognizable by untrained panelist; (d) 3.5 < ΔE < 5—a visible color difference; (e) 5 < ΔE—impression of two different colors [61]. The obtained ΔE value differences indicate that ice cream color changes could be identified by untrained panelists, especially if the mineral source concentration exceeds 0.8%. Moreover, the ice cream exhibited a dose-dependent color saturation (C*) change, which is considered as a parameter for quantitative color attributes perceived by people [62]. Storage of the ice cream resulted in overall color changes in all tested samples, however, the greatest were observed in ice cream with a 1.0% mineral complex addition (Table 5). 

#### 3.2.2. Ice Cream Overrun

**Figure 2:**
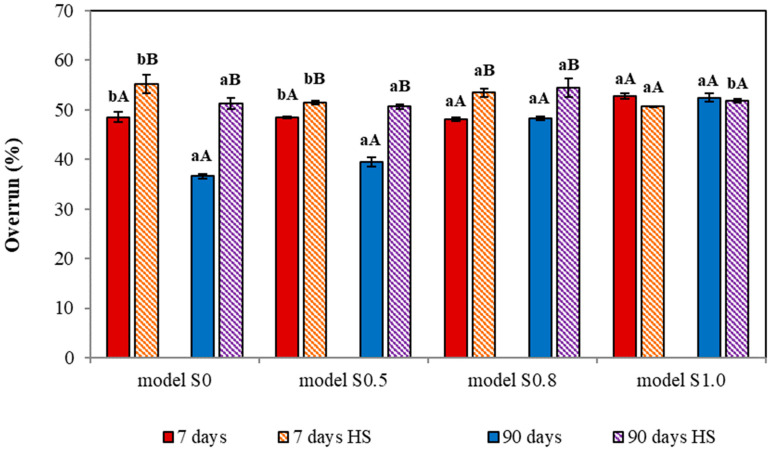
Ice cream overrun after production, frozen storage, and temperature fluctuations; HS—heat shock. a, b—values with different letters (within the same model) of different storage times are significantly different (α < 0.05). A, B—values with different capital letters (within the same model) of different temperature conditions are significantly different (α < 0.05).

The next step of the study was the analysis of ice cream overrun. Air is a crucial component responsible for ice cream structure development. The quantity of air incorporated during freezing determines the size of ice crystals and the larger crystals are observed in ice cream with lower overrun [63]. In addition, the relationship between formulations’ apparent viscosity and smaller air cells in ice cream structure has been proven by Chang and Hartel (2002) [64]. Overrun is reported to impact the ice cream consistency and its physical properties like melting rate [65]. In the presented study, the ice cream overrun was evaluated after (a) production (7 days), (b) 90 days frozen storage, and (c) heat shock applications. The obtained results are presented in the figure below (Figure 2).

The reference samples and the low mineral ice cream samples (S0.5; S0.8) were characterized by similar average overrun values (48.06 ± 0.36% to 48.55 ± 1.08%), which were not significantly different. Higher overrun values were observed in ice cream with 1.0% multimineral preparation, which could be a result of smaller air bubbles formation during freezing. This could suggest that ice cream with lower sucrose content and higher minerals content showed greater fluffiness when compared to the reference. The 90-day frozen storage resulted in an overrun decrease, which was considered significant in models S0 and S0.5. Lower overrun values after storage were observed in research performed for a variety of ice creams. Güzeler et al. (2012) claimed that the stabilizer and emulsifier ratio is not a determinant factor for ice cream overrun, however, the storage time is [66]. After 30 days, the air content in milk ice cream was lower by 1.00–10.75% and these values increased after consecutive storage to 1.50–13.75% (60-day period). Similar results were obtained by Murtaza et al. (2004) in milk ice cream where the overrun changed from 56.57 to 53.00% after 40 days storage time [67]. According to Singh et al. (2014), the progress in storage time caused a gradual decrease in flavored ice cream overrun which, after 30 days, was around 1.30–1.70% [68]. In our study the extent of overrun decrease was in the range of 0.28 to 11.95%. The relation between overrun, air bubble size, and ice crystallization has been studied and well described in the literature, although it is not fully understood. Sofjan and Hartel (2004) reported that increased ice cream overrun leads to smaller air cells and ice crystal formation [69]. Similar conclusions were presented in the study by Warren and Hartel (2018) and VanWess et al. (2019) [70,71]. In addition, the storage time, especially in the temperature range from −30.0 to −18.0 °C, promotes air cell coalescence and ice crystal development [65,72]. 

In general, after the application of thermal fluctuations, the higher overrun values were observed in all the ice cream samples. Ice creams after the production subjected to thermal stress were characterized with similar overrun values both in samples after the production and those stored for 90 days. The overrun ranged from 50.65 ± 0.17 to 55.25 ± 1.91% and 50.65 ± 0.47 to 54.39 ± 1.88%, respectively (Figure 2). The probable cause of increased overrun values might be related to the ice cream structural changes and the measurement difficulties. Temperature fluctuations, the increase in temperature in particular, results in ice crystals melting and water compounds’ movement. This leads to unfrozen water mobility intensification and the coalescence of air cells within the ice cream, which had been observed especially in samples stored at high temperatures (−10.0 or −6.0 °C) [69]. Elevated temperatures can facilitate the upward movement of air cells to the outer surface of the ice cream, slightly altering its structure. This is of particular importance with respect to the sample area taken for analysis, as it may partially interfere with overrun results. The subsequent rapid decrease in the temperature causes water freezing and ice recrystallization, often into ice crystals larger than before the thermal stress [73]. Nevertheless, the ice crystal formation phenomenon depends not only on the freezing process and storage conditions but also on formulation characteristics, thus the stabilizing and cryoprotective substances addition [8,73,74]. In the presented work, the addition of mineral algae complex, especially in 1.0% concentration, appeared to have a relatively similar preserving effect on ice cream overrun both in samples without and after the thermal treatment. Unfortunately, we have not encountered studies concerning the direct impact of thermal fluctuations on ice cream overrun, hence comparison of our results with the literature was impossible.

#### 3.2.3. Ice Cream Meltdown

**Figure 3:**
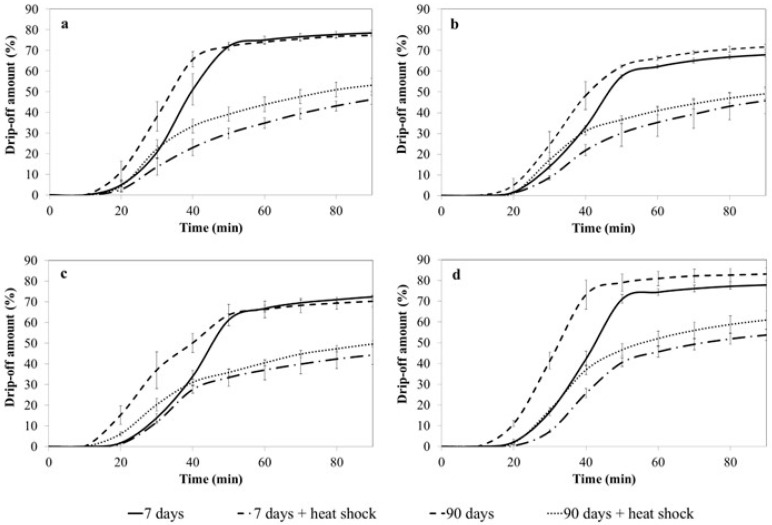
Melting curves of four ice cream models before and after thermal treatment: (**a**)—model S0 (reference sample), (**b**)—model S0.5, (**c**)—model S0.8, (**d**)—model S1.0.

The chemical composition of ice cream formulations and the physical structure of the ice cream itself affect the melting dynamics. In the present research, four different ice cream models were analyzed for their melting behavior by monitoring the amount of drip-off over time and the occurrence of the first drop. The samples were examined after the selected storage time and after the thermal stress. The melting curves for all the ice cream models tested under different storage conditions are presented in Figure 3.

It was observed that freshly made ice cream (7 days storage) had a very similar melting behavior. The first drop was observed after 14–15 min of the analysis. After 90 min, the amount of drip-off material was in the range of 68.01–78.48%, with the greatest drip-off found in the reference model (78.48%) and model S1.0 (77.87%). Although the ice melted completely, the ice cream structure did not collapse, which was visible in the form of foam that remained on the surface of the metal wire mesh. This material represented approximately 20.5 to 40.00% of the initial ice cream weight and was considered to be the residual network of macromolecules responsible for maintaining the ice cream structure. The melting behavior of all ice cream models tested changed significantly after the application of thermal fluctuations. The first drop in the reference ice cream sample was observed earlier than in the unstressed samples. On the contrary, the first drop in the ice cream with multimineral preparation addition appeared about 2.5–4.2 min later, independent of the dose of multimineral preparation. Interestingly, the final amount of drip was about 50.00% of the initial weight of the ice cream, which was significantly lower than in samples not subjected to thermal stress. 

The 90-day frozen storage time did not significantly change the final drip-off amount of the ice cream, although more intense melting of the ice cream was observed. This was shown by a shift in the melting curves (Figure 3). The first drop of the reference ice cream samples and the 0.5% marine algae complex samples was observed after 14 min of incubation, thus was similar to freshly made ice cream. Surprisingly, the first drop appeared much earlier (about 11 min) in samples with 0.8% and 1.0% addition of the multimineral preparation. The application of thermal fluctuations resulted in a similar effect as in the previous experiment. The final drip-off amount was 50.0–60.0% of the initial ice cream weight, with the residue which did not collapse and remained on the surface of the metal wire mesh. The stressed ice cream samples melted less rapidly than the untreated samples (Figure 3).

Several researchers claim that the modification of ice cream formulation ingredients and freezing process parameters affects the formation of ice cream microstructure and thus its physical parameters [51,71,75,76]. Many studies indicate that ice cream with higher overrun melts more slowly than those with low overrun, likely due to the slowed heat transfer through the air cells [69,70,71,73]. Moreover, melting behavior can provide additional information about the internal structure of the ice cream. According to Wu et al. (2019), ice cream melting occurs either as a complete drip-through of the serum phase or as a gradual decrease in ice cream height, leaving a residual foam [76]. The latter was observed in our study for all ice cream variants tested, although the weight of the remaining ice cream material differed significantly. The ice cream stored under proper conditions dripped at around 70.00–84.00%, while a lower drip amount was observed in the samples subjected to thermal stress (Figure 3). Thus, the improper storage conditions affect the microstructure of the ice cream and alter the melting process. Similar conclusions were made by Lomolino et al. (2020) in the study of dairy and vegan ice cream [73]. Furthermore, Park et al. (2015) claim that the melting behavior of ice cream differs depending on the storage temperatures, especially below −30.00 °C [65].

**Figure 4:**
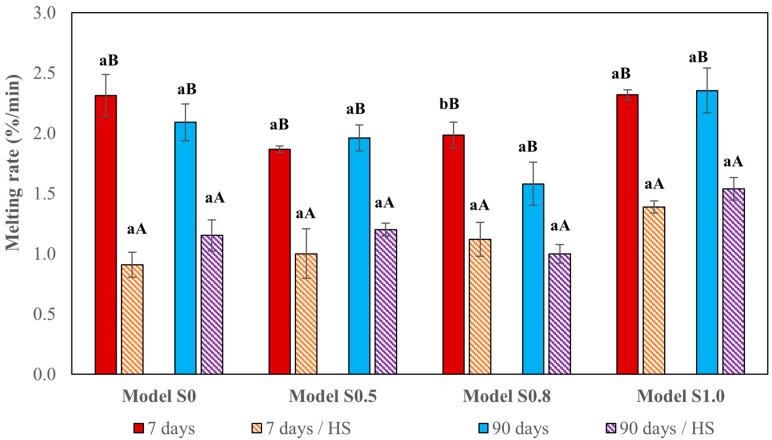
Comparison of ice cream melting rates after the frozen storage and thermal stress; HS—heat shock. a, b—values with different letters (within the same model) of different storage times are significantly different (α < 0.05). A, B—values with different capital letters (within the same model) of different temperature conditions are significantly different. (α < 0.05).

Indeed, in our study, ice cream models showed changes in their melting behavior during the treatment; however, the storage time seemed not to affect the ice cream condition. The melting rates of all ice cream models both after 7 and 90 days of frozen storage were similar, with the highest values in model S1.0 ice cream (Figure 4). A significant difference was noted for model S0.8 ice cream only. Thermal fluctuations influenced ice cream physicochemical features and consequently resulted in melting rate changes in all studied ice cream samples. Not only did ice cream melting decelerate in samples stored for 7 days and in samples stored for 90 days, but also melting curve flattening was observed (Figure 3 and Figure 4). 

Ice cream formulation composition and freezing process determine the final ice cream structure. It was proven that ice cream with milk components (proteins, fats) melted slower than plant-based ice cream or ice cream supplemented with inulin [73,77,78]. The addition of other components, e.g., Moldavian balm, grape wine lees, chia seed mucilage, mushroom powder, or hydrocolloids might limit the melting process as well [79,80,81,82]. According to Muse and Hartel (2004) and Park et al. (2015), a uniform distribution of small ice crystals and air cells facilitates heat transfer reduction and hence decreases the melting rate [22,65]. Furthermore, some ice creams with lower overrun were found to express higher melting rates after storage at −30.00 °C [83]. In addition, many researchers claim that partially coalesced fat has the greatest impact on meltdown behavior; however, further investigation concerning the relationship between meltdown behavior and fat destabilization in different ice cream models is still required [51]. 

#### 3.2.4. Texture Analysis

Many factors, including formulation composition, viscosity, ice crystals size, and, overrun, determine the texture of ice cream. The hardness of the ice cream samples tested varied among the models tested and storage conditions. Ice cream supplemented with 1.0% multimineral preparation expressed significantly higher values of texture and adhesiveness parameters. The texture of the reference sample and the ice cream with 0.5% mineral addition was similar (Table 6). Frozen storage for 90 days resulted in an increase of the hardness in all tested samples except model S1.0. Both texture and adhesiveness were lower for model S1.0 ice cream than before storage. The addition of minerals did not appear to have a major effect on ice cream texture parameters. The ice cream samples had relatively similar hardness, so the addition of minerals did not increase the fragility of ice cream, and the fluffiness did not seem to be affected. Of note are the results for ice cream subjected to thermal treatment. Freshly made ice cream samples were observed to express higher hardness parameters than untreated samples, but only the results of model S1.0 ice cream differed significantly. On the contrary, ice cream stored for 90 days and subjected to thermal stress had lower hardness and adhesiveness parameters. Therefore, it appeared that the effect of mineral preparation on the ice cream texture was negligible.

Many studies claim that ice cream hardness varies depending on the formulation composition and, therefore, the physicochemical characteristics of ice cream. The overrun is considered as a parameter responsible for ice cream hardness as well, however, the literature data are ambiguous [69,84,85,86]. It was stated by Syed et al. (2018) that ice cream hardness is rather affected by emulsifiers than the type of milk fat added [83]. According to Muse and Hartel (2004), milk-based ice creams’ texture depends on the carbohydrate source [22]. The greatest hardness was noted for ice cream containing high fructose corn syrup whereas sucrose-based ice cream was assigned as intermediate hardness. In our study, ice cream models made with different percentage ratios of sucrose and multimineral preparation (10.0:0, 9.5:0.5, 9.2:0.8, and 9.0:1.0 for models S0, S0.5, S0.8, and S1.0, respectively) exhibited greater hardness with higher mineral component concentration.

#### 3.2.5. Sensory Evaluation

**Figure 5:**
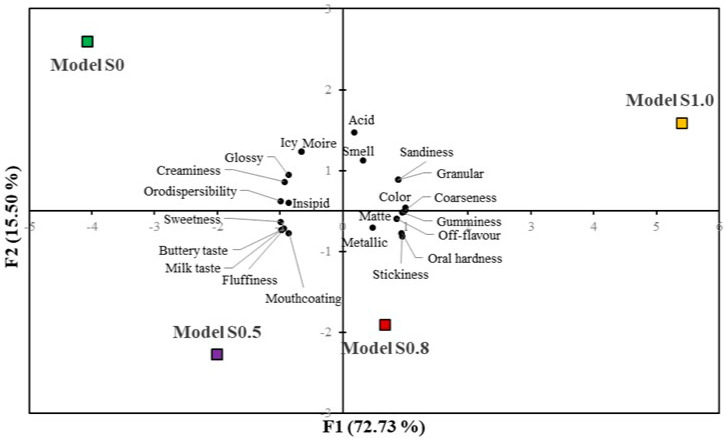
Principal component analysis of the matrix of mean attributes rating across ice cream samples.

Principal component analysis was performed to demonstrate the sensory evaluation of ice cream with the addition of multimineral preparation. The primary component (F1) explained 72.73% of the total variance and was associated mainly with ice cream textural features including gumminess (0.947), coarseness (0.999), dispersibility (−0.991), as well as the color (0.997) and sweetness (−0.990). The second major component (F2) explained 15.50% of the total variance and was rather associated with appearance and taste characteristics (Figure 5). 

According to the results, the investigated samples expressed differentiated properties despite similarities in sense profiling scoring. Ice cream with none and 0.5% marine algae complex content was creamy with a relatively high orodispersible structure. The sensory assessment revealed that model S0 and S0.5 ice cream expressed good mouthcoating features which correlated with the degree of fluffiness, although the overrun values for models S.0, S0.5, and S0.8 did not differ significantly. The increase in marine algae preparation content favored the increase of coarseness, gumminess, and overall oral hardness. According to panelists, the ice cream seemed to possess a more sandy and granular structure, in particular in model S1.0. These results are consistent with texture measurement where model S1.0 ice cream hardness was found to be nearly two times greater than for the reference sample. Both model S0 and model S0.5 ice cream were assigned as highly sweet with a milky and buttery taste. Ice cream with a 1.0% mineral complex appeared less sweet with a slightly metallic taste. These results could be explained by the differentiated ice cream mixes composition. While all ice cream models had the same amount of skim milk powder added, the amounts of sugar and multimineral preparation were inversely proportional. Therefore, the samples with the lowest sugar content had the highest mineral content, which could cause the metallic taste and at the same time partially suppress the sweetness. A similar change in taste perception was observed in the study of ice cream with different ratios of sucrose and grape pulp and skin matter [87]. Model S0 ice cream exhibited a moire but glossy surface structure which was not observed in ice cream with 0.8% mineral preparation, whereas both model S0.8 and model S1.0 ice cream were characterized by matte and light yellowish color. Some sensory properties were evaluated together and their average scoring values are given in Table 7. Taking into consideration the personal preferences of the assessors, mean values were calculated for appearance, texture, and flavor. 

According to the sense profiling, the texture and mouthfeel were similar in all studied ice cream models. Significant differences were identified in the appearance and taste features. The results show that ice cream S0.8 (0.8% marine algae complex) scored the best in overall acceptability among all tested models (Table 8). The degree of sweetness and metallic aftertaste were perceived as the most balanced, although the milk and buttery taste were less noticeable. These results are promising for the development of ice cream products that are acceptable to consumers and that could also retain functional food properties when enriched with functional nutrients. Tsai et al. (2020) came to a similar conclusion when they investigated milk ice cream with the addition of silver ear mushroom powder [81].

#### 3.2.6. Nutrient Density

Describing the nutritional quality of foods is of great importance when dealing with dietary guidelines and developing diets. Nutrient profiling indices, which are science-based attempts to capture the multiple nutritional attributes of foods, help individuals choose and consume healthier diets. Nutrient density is estimated by nutrient profiling methods and generally refers to the specific nutrient content per product portion (100 g, 100 kcal, per serving). Currently, the term “nutrient-dense” is associated with foods that are rich in health-promoting nutrients and low in dietary energy.

Marine algae and plants are known to be rich in bioactive compounds such as fat- and water-soluble vitamins and essential microelements, which can influence food properties and functionality [88]. In our research, the dairy ice cream was enriched with the multimineral preparation from Atlantic red algae, which is rich in nutrients, so its addition was intended both to supplement some essential microelements and to increase the nutritional density of the ice cream produced. This was successfully accomplished (Table 8). In order to maintain a constant dry matter content in the ice cream models tested, a higher amount of multimineral preparation was balanced with a lower amount of sucrose, which affected the calculated energy density and had an impact on the final results.

**Table 8:** Ice cream nutrient density for the selected indices.

Indices	Model			
	S0	S0.5	S0.8	S1.0
Naturally Nutrient-Rich Score (NNR)				
NNR14	87.21	113.62	129.78	140.69
NNR16	83.39	106.59	120.79	130.36
Nutrient-Rich Foods (NRF)				
NRF6.3	0.51	18.45	29.42	36.83
NRF9.3	9.44	30.08	42.70	51.23
NRF11.3	17.99	38.79	51.51	60.09
NRF15.3	38.25	56.75	68.06	75.69
Nutrient Adequacy Score (NAS)				
NAS	6.63	8.59	9.77	10.56
Nutrient Density Score (NDS)				
NDS5	4.43	8.02	10.21	11.69
NDS6	5.58	8.26	9.90	11.01
NDS9	4.51	6.81	8.22	9.18
NDS16	4.21	5.52	6.32	6.86

Dietary Reference Values for EU (EFSA) were taken for the calculation of indices (adults, both genders, sedentary lifestyle) [89] (https://efsa.gitlab.io/multimedia/drvs/index.htm, accessed on 4 May 2023).

Nutrient profiling results based on Naturally Nutrient-Rich (NNR), Nutrient Adequacy Score (NAS), and Nutrient Density Score (NDS) assessments showed that the nutrient density of the fortified ice cream models increased significantly. In addition, the index values were two times higher in samples with a 1.0% mineral content than in the reference model. More differentiated results were obtained according to the Nutrient-Rich Food Index (NRF). Ice cream is one of the most frequently chosen snacks, so modifying its compound ratio may improve the nutritional value of the diet [90], especially by reducing the carbohydrate content while increasing the amount of microelements. This was demonstrated for ice cream enriched with peach fiber, but the change in organoleptic characteristics was also noted [33]. In our study, the ice cream with 1.0% multimineral preparation was characterized by a high nutritional value, but the panelists claimed to perceive a slightly metallic off-taste which lowered the organoleptic acceptance. This was not observed in the S0.8 ice cream, which also had a relatively high nutritional value. In addition, the composition of the multimineral preparation contained relatively high levels of calcium, which is considered an important element for human health. Ferrar et al. (2010) reported that daily consumption of calcium-enriched ice cream for 28 days could have a beneficial effect on bone mass, especially in premenopausal women who do not meet the recommended calcium intake [91]. 

## 4. Summary and Conclusions

In this research, the effect of a multimineral preparation from the Atlantic red algae *Lithothamnion* sp. on the properties of milk fat ice cream was investigated. It was found that the addition of selected sucrose and multimineral preparation ratios slightly but not significantly affected the freezing process and dynamics by increasing the glass transition and freezing temperatures. According to our results, the ice cream formulations tested exhibited a typical non-Newtonian pseudoplastic flow type. The apparent viscosity values of ice cream decreased with lower sucrose and higher mineral content. It was concluded that selected mineral contents (0.5, 0.8, and 0.1%) in ice cream did not significantly affect the rheological properties. The tested mineral contents had a weak effect on the physicochemical and thermophysical ice cream properties, even after temperature treatment. The overrun values in ice cream supplemented with 0.8% and 1.0% mineral preparation were maintained at a similar level, both after the frozen storage and after thermal fluctuation. The resistance to melting increased slightly in fresh and stored ice cream, except for the 1.0% samples. A similar trend was observed for drip-off measurements and after thermal fluctuations. The multimineral preparation slightly increased the ice cream texture parameters, which seemed to remain relatively stable after thermal stress. Therefore, the addition of the presented mineral contents does not increase the fragility of the ice cream and does not affect its fluffiness. According to the organoleptic evaluation, the reference samples and the samples with a low (0.5%) content of the mineral complex had a creamier structure, better orodispersibility and mouthcoating properties, a glossy appearance, and had a typical milky and buttery taste. A high concentration of the preparation left a slightly metallic aftertaste. On the other hand, a higher content of marine algae preparation resulted in a higher nutrient density and a lower energy value. 

The abovementioned issues could lead the authors to the conclusion that such small changes in multimineral preparation and sucrose content do not alter the major milk fat ice cream characteristics, predominantly freezing process, rheological parameters, overrun, and texture. In fact, 0.8 and 1.0% mineral concentrations appear to have a moderate preservative effect on ice cream overrun, especially after thermal shock. Therefore, it can be suggested that higher mineral content may have a weak cryoprotective effect on ice cream overrun or structure-forming ability, as well as the quality during frozen storage. The addition of minerals allows to increase the nutrient density and nutritional value of the ice cream and could provide the supplementation of macro- and microelements which are important for human nutrition, while following the health-promoting aspect of functional food. However, the amount of the minerals added should be monitored in order to overcome problems associated with off-taste, off-flavor, and changes in the texture of the ice cream. Therefore, the addition of 0.8% Atlantic red algae multimineral preparation seemed to be the most promising in terms of sensory acceptance by consumers and variations in physicochemical properties that are of high importance for technological processing and ice cream production. However, further studies on sugar and milk fat replacers in ice cream with high nutrient and energy value are required.

## Figures and Tables

**Table 1:** Composition of mixes used for ice cream production.

Ingredients [%]	Ice Cream Mix Model			
	S0	S0.5	S0.8	S1.0
Sucrose	10.00	9.50	9.20	9.00
Skim milk powder	12.00	12.00	12.00	12.00
Milk fat	8.00	8.00	8.00	8.00
Palsgaard 260	0.50	0.50	0.50	0.50
Multi-mineral preparation	0	0.50	0.80	1.00
Water	69.50	69.50	69.50	69.50

**Table 2:** Ice cream formulations’ physical parameters.

Parameter		Model			
		S0	S0.5	S0.8	S1.0
Dry mass	(g)	32.56 ± 0.17 ^a^	32.54 ± 0.08 ^a^	32.51 ± 0.05 ^a^	32.52 ± 0.07 ^a^
a_w_	(-)	0.9851 ± 0.0004 ^a^	0.9853 ± 0.0003 ^a^	0.9853 ± 0.0003 ^a^	0.9852 ± 0.0004 ^a^
pH	(-)	6.58 ± 0.04 ^a^	6.99 ± 0.04 ^b^	7.05 ± 0.06 ^b^	7.10 ± 0.04 ^b^
ED	kcal	157.61 ^a^	155.72 ^ab^	154.58 ^b^	153.82 ^b^
	kJ	659.30 ^a^	651.25 ^a^	646.42 ^b^	643.20 ^b^

where: a_w_—water activity; ED—energy density; values in rows with the same index letter are not significantly different from each other at α < 0.05.

**Table 3:** The values determined by DSC analysis in ice cream samples.

Model			S0	S0.5	S0.8	S1.0
Melting reaction	Temperature (°C)	Tf	−1.74 ± 0.14 ^a^	−1.64 ± 0.08 ^b^	−1.61 ± 0.05 ^b^	−1.63 ± 0.07 ^b^
		onset	−8.1 ± 0.20 ^a^	−5.6 ± 0.30 ^b^	−5.4 ± 0.20 ^b^	−5.3 ± 0.20 ^b^
		offset	3.4 ± 0.30 ^a^	8.5 ± 0.20 ^b^	9.0 ± 0.20 ^b,c^	9.3 ± 0.20 ^c^
		ΔT (°C)	11.5 ^a^	14.1 ^b^	14.4 ^b^	14.6 ^b^
	Parameter	ΔH (J/g)	221.80 ± 6.41 ^a^	217.03 ± 3.22 ^a^	216.36 ± 2.14 ^a,b^	209.96 ± 3.31 ^b^
		FW (%)	66.41 ± 0.14 ^a^	64.97 ± 0.07 ^b^	64.78 ± 0.04 ^b^	62.88 ± 0.10 ^c^
		MC (%)	68.53 ± 0.14 ^a^	68.16 ± 0.14 ^a^	68.21 ± 0.08 ^a^	67.65 ± 0.14 ^b^
		UFW (%)	2.12 ± 0.14 ^a^	3.19 ± 0.14 ^b^	3.43 ± 0.14 ^c^	4.79 ± 0.14 ^d^
Glass transition (°C)		Tg	−47.28 ± 0.10 ^a^	−46.54 ± 0.20 ^a^	−46.40 ± 0.20 ^a^	−46.26 ± 0.30 ^a^
		onset	−49.36 ± 0.10 ^a^	−48.66 ± 0.20 ^a^	−48.26 ± 0.20 ^a^	−49.25 ± 0.40 ^a^

where: Tf—freezing temperature; ΔH—endothermic enthalpy of the melting; FW—freezable water (ice formed); MC—moisture content; UFW—unfreezable (bound) water; Tg—glass transition temperature; values in rows with the same index letter are not significantly different from each other at α < 0.05.

**Table 4:** Rheological parameters of ice cream mixes.

Ice Cream Mix	Apparent Viscosity *	Model						
		Ostwald de Waele			Herschel–Bulkley			
		k (Pa·s^n^)	n (-)	R^2^	*σ*_0_ (Pa)	k (Pa·s^n^)	n (-)	R^2^
S0	0.0677 ± 0.0005 ^c^	1.0000 ^a^	0.5414 ^c^	0.9971 ^a^	1.7842 ^c^	0.2619 ^a^	0.7549 ^a^	0.9997 ^a^
S0.5	0.0652 ± 0.0004 ^b^	1.0000 ^a^	0.5353 ^b^	0.9962 ^a^	1.0241 ^a^	0.2831 ^a^	0.7459 ^a^	0.9997 ^a^
S0.8	0.0621 ± 0.0005 ^a^	1.0000 ^a^	0.5286 ^a^	0.9961 ^a^	1.2332 ^b^	0.2544 ^a^	0.7504 ^a^	0.9997 ^a^
S1.0	0.0637 ± 0.0007 ^a^	1.0000 ^a^	0.5312 ^a,b^	0.9963 ^a^	1.7661 ^c^	0.2293 ^a^	0.7667 ^a^	0.9997 ^a^

* apparent viscosity measured at 350.00 s^−1^ shear rate; values in columns with the same index letter are not significantly different from each other at α < 0.05.

**Table 5:** Color parameters of ice cream models supplemented with marine algae complex.

Ice Cream Model	Time [days]	Color Parameter					
		L*	a*	b*	C*	h	ΔE
S0	7	89.64 ± 0.21 ^c^	−0.33 ± 0.03 ^d^	13.20 ± 0.13 ^c^	13.21 ± 0.13 ^c^	91.43 ± 0.13 ^b^	0 ^c^
S0.5		88.29 ± 0.41 ^a^	−0.56 ± 0.02 ^a,b,c^	12.00 ± 0.59 ^a^	12.01 ± 0.60 ^a^	92.66 ± 0.10 ^a^	1.92 ± 0.27 ^a,b^
S0.8		88.19 ± 0.48 ^a,b^	−0.51 ± 0.01 ^a,b^	11.37 ± 0.35 ^a,b^	11.38 ± 0.35 ^a,b^	92.59 ± 0.16 ^a^	2.35 ± 0.57 ^b^
S1.0		87.38 ± 0.12 ^a^	−0.51 ± 0.02 ^a,b^	10.63 ± 0.24 ^b^	10.64 ± 0.24 ^b^	92.75 ± 0.08 ^a^	3.43 ± 0.19 ^d^
S0	90	87.95 ± 0.82 ^a^	−0.51 ± 0.09 ^b^	12.96 ± 0.17 ^c^	12.97 ± 0.17 ^c^	92.24 ± 0.40 ^c^	1.71 ± 0.29 ^a^
S0.5		87.83 ± 0.21 ^a^	−0.60 ± 0.03 ^c^	12.04 ± 0.13 ^a^	12.06 ± 0.12 ^a^	92.87 ± 0.12 ^a^	2.17 ± 0.17 ^a,b^
S0.8		88.48 ± 0.17 ^a,b^	−0.57 ± 0.03 ^a,c^	11.75 ± 0.11 ^a,b^	11.76 ± 0.11 ^a,b^	92.76 ± 0.17 ^a^	1.87 ± 0.08 ^a,b^
S1.0		89.55 ± 0.16 ^b,c^	−0.54 ± 0.01 ^a,b^	8.78 ± 0.08 ^d^	8.80 ± 0.08 ^d^	93.51 ± 0.08 ^d^	4.43 ± 0.08 ^e^

L*—brightness from 0 (black) to 100 (white), a* yellowness; b* redness; C* color saturation, h color tone; ΔE total color difference. values in columns with the same index letter are not significantly different from each other at α < 0.05.

**Table 6:** Comparison of texture and adhesiveness parameters of prepared ice cream models.

		Hardness, T [N]		Adhesiveness, A [mJ]	
Ice Cream Model *	Days	Before Heat Shock	After Heat Shock	Before Heat Shock	After Heat Shock
S0	7	1.68 ± 0.18 ^a,A^	1.88 ± 0.43 ^a,A^	2.47 ± 0.37 ^x,X^	2.17 ± 0.58 ^x,X^
S0.5		1.69 ± 0.17 ^a,A^	1.91 ± 0.22 ^a,A^	2.17 ± 0.58 ^x,X^	1.81 ± 0.19 ^x,X^
S0.8		2.09 ± 0.09 ^a,A^	2.20 ± 0.17 ^a,A^	2.17 ± 0.42 ^x,X^	2.70 ± 0.22 ^y,X^
S1.0		3.86 ± 0.43 ^b,B^	2.42 ± 0.25 ^a,A^	3.90 ± 0.40 ^y,Y^	3.00 ± 0.49 ^x,X^
S0	90	2.36 ± 0.54 ^a,B^	2.00 ± 0.18 ^a,A^	3.20 ± 0.33 ^y,Y^	2.52 ± 0.32 ^x,X^
S0.5		2.28 ± 0.20 ^a,B^	2.12 ± 0.32 ^a,A^	2.25 ± 0.36 ^x,X^	2.33 ± 0.36 ^x,Y^
S0.8		2.52 ± 0.20 ^a,B^	2.27 ± 0.27 ^a,A^	2.67 ± 0.47 ^x,X^	2.45 ± 0.28 ^x,X^
S1.0		2.72 ± 0.12 ^b,A^	2.37 ± 0.18 ^a,A^	3.22 ± 0.62 ^x,X^	2.75 ± 0.24 ^x,X^

* results present the average values of the attributes using a 1–9 point rating scale; ^a, b, x, y^,—values with different letters within the rows (same model) are significantly different (α < 0.05). ^A, B, X^, and ^Y^—values with different capital letters within the columns (same temperature treatment) are significantly different (α < 0.05).

**Table 7:** Results of ice cream sensory assessment.

Ice Cream Model *	Color and Appearance	Texture/Mouthfeel	Odor and Flavor	Overall Acceptability
S0	3.27 ± 0.25 ^b^	3.95 ± 0.15 ^a^	4.29 ± 0.25 ^a^	6.67 ± 0.47 ^b^
S0.5	3.37 ± 0.28 ^b^	3.85 ± 0.24 ^a^	4.23 ± 0.23 ^a^	6.33 ± 0.47 ^b^
S0.8	3.53 ± 0.25 ^b^	3.98 ± 0.20 ^a^	4.06± 0.30 ^a^	7.50 ± 0.50 ^a^
S1.0	4.00 ± 0.22 ^a^	3.88 ± 0.18 ^a^	3.73± 0.29 ^b^	6.67 ± 0.75 ^b^

* results present the average values of the attributes using a 1–9 point rating scale; ^a, b^—values with different letters within the columns are significantly different (α < 0.05).

## Data Availability

The key data generated and used to support the findings of the study are presented in the article. The remaining detailed datasets are available from the corresponding author upon reasonable request.

## Supplementary Materials

The following supporting information can be downloaded at: https://www.mdpi.com/article/10.3390/foods12244481/s1. Table S1: Multimineral preparation trace mineral composition.
