# Supplementary material for: The Physicochemical Properties and Melting Behavior of Ice Cream Fortified with Multimineral Preparation from Red Algae
Source: Foods. 2023 Dec 14;12(24):4481. doi: 10.3390/foods12244481 (PMC10742974; doi:10.3390/foods12244481)
Supplement: Supplementary file 1 [file foods-12-04481-s001.zip › foods-2707097-supplementary.pdf]

Supplementary Materials

Table S1. Multimineral preparation trace mineral composition.

Component	Amount	Unit	Component	Amount	Unit
Aluminum	79.50	ppm	Manganese	64.60	ppm
Antimony	2.99	ppm	Mercury	<0.001	ppm
Arsenic	1.265	ppm	Molybdenum	2.10	ppm
Barium	7.22	ppm	Neodymium	0.387	ppm
Beryllium	1.83	ppm	Nickel	3.28	ppm
Bismuth	<0.50	ppm	Niobium	<0.50	ppm
Boron	42.50	ppm	Palladium	0.408	ppm
Cadmium	0.659	ppm	Phosphorus	83.70	ppm
Calcium	33.70	%	Platinum	0.002	ppm
Carbon	125,000	ppm	Potassium	179.00	ppm
Cerium	0.700	ppm	Praseodymium	0.087	ppm
Cesium	<0.001	ppm	Rhenium	0.002	ppm
Chloride	38.00	ppm	Rhodium	0.185	ppm
Chromium	3.39	ppm	Rubidium	0.010	ppm
Cobalt	2.93	ppm	Ruthenium	2.284	ppm
Copper	3.56	ppm	Samarium	0.090	ppm
Dysprosium	0.116	ppm	Scandium	0.928	ppm
Erbium	0.083	ppm	Selenium	<0.50	ppm
Europium	0.030	ppm	Silicon	27.50	ppm
Fluoride	0.18	ppm	Silver	3.01	ppm
Gadolinium	0.115	ppm	Sodium	4,014	ppm
Gallium	0.162	ppm	Strontium	1,947	ppm
Germanium	<0.001	ppm	Sulfur	3,916	ppm
Gold	3.05	ppm	Tantalum	<0.001	ppm
Hafnium	<0.001	ppm	Tellurium	5.47	ppm
Holmium	0.026	ppm	Terbium	0.020	ppm
Indium	0.13	ppm	Thallium	<0.50	ppm
Iodine	0.13	ppm	Thorium	2.23	ppm
Iridium	0.001	ppm	Thulium	0.011	ppm
Iron	1,007	ppm	Tin	0.056	ppm
Lanthanum	<0.50	ppm	Titanium	35.70	ppm
Lead	0.038	ppm	Tungsten	<0.50	ppm
Lithium	6.28	ppm	Vanadium	9.30	ppm
Lutetium	0.025	ppm	Yttrium	4.82	ppm
Osmium	0.002	ppm	Zinc	3.56	ppm
Magnesium	3.02	%	Zirconium	<0.50	ppm

Article Title: The Physicochemical Properties and Melting Behavior of Ice Cream Fortified with Multimineral Preparation from Red Algae

Journal name: Foods, MDPI

The Authors: Markowska Joanna [1,*], Tyfa Agnieszka [1], Drabent Anna [1], Stępniak Artur [2]

[1] Prof. Waclaw Dabrowski Institute of Agricultural and Food Biotechnology—State Research Institute (IBPRS-PIB), Department of Refrigeration Technology and Technique, Marszalka J. Pilsudzkiego 84 Avenue, 92-202 Lodz, Poland

[2] Unit of Biophysical Chemistry, Department of Physical Chemistry, Faculty of Chemistry, University of Lodz, Pomorska 163/165 Street, 90-236 Lodz, Poland

* Correspondence: joanna.markowska@ibprs.pl
